# Transport and Secretion of the Wnt3 Ligand by Motor Neuron-like Cells and Developing Motor Neurons

**DOI:** 10.3390/biom11121898

**Published:** 2021-12-17

**Authors:** Cristina Pinto, Viviana Pérez, Jessica Mella, Miguel Albistur, Teresa Caprile, Francisca C. Bronfman, Juan Pablo Henríquez

**Affiliations:** 1 Neuromuscular Studies Laboratory (NeSt Lab), CMA Bio-Bio, Group for the Study of Developmental Processes (GDeP), Department of Cell Biology, Universidad de Concepción, Concepción 4070112, Chile; cristinapinto@udec.cl (C.P.); viviperez@udec.cl (V.P.); jemella@udec.cl (J.M.); miguelalejalbis@udec.cl (M.A.); 2 Axon Guidance Laboratory, Group for the Study of Developmental Processes (GDeP), Department of Cell Biology, Universidad de Concepción, Concepción 4070112, Chile; tcaprile@udec.cl; 3 Institute of Biomedical Sciences (ICB), Faculty of Medicine and Faculty of Life Science, Universidad Andres Bello, Santiago 8320000, Chile; francisca.bronfman@unab.cl; 4 CARE Biomedical Research Center, Pontificia Universidad Católica de Chile, Santiago 8320000, Chile

**Keywords:** Wnt, motor neuron, neuromuscular junction

## Abstract

The vertebrate neuromuscular junction (NMJ) is formed by a presynaptic motor nerve terminal and a postsynaptic muscle specialization. Cumulative evidence reveals that Wnt ligands secreted by the nerve terminal control crucial steps of NMJ synaptogenesis. For instance, the Wnt3 ligand is expressed by motor neurons at the time of NMJ formation and induces postsynaptic differentiation in recently formed muscle fibers. However, the behavior of presynaptic-derived Wnt ligands at the vertebrate NMJ has not been deeply analyzed. Here, we conducted overexpression experiments to study the expression, distribution, secretion, and function of Wnt3 by transfection of the motor neuron-like NSC-34 cell line and by in ovo electroporation of chick motor neurons. Our findings reveal that Wnt3 is transported along motor axons in vivo following a vesicular-like pattern and reaches the NMJ area. In vitro, we found that endogenous Wnt3 expression increases as the differentiation of NSC-34 cells proceeds. Although NSC-34 cells overexpressing Wnt3 do not modify their morphological differentiation towards a neuronal phenotype, they effectively induce acetylcholine receptor clustering on co-cultured myotubes. These findings support the notion that presynaptic Wnt3 is transported and secreted by motor neurons to induce postsynaptic differentiation in nascent NMJs.

## 1. Introduction

The vertebrate neuromuscular junction (NMJ) allows muscle contraction and controls the coordinated movement of organisms. The NMJ is composed by a presynaptic motor nerve terminal, a postsynaptic muscle specialization, and non-myelinating terminal Schwann cells (tSC) [1,2]. During embryonic NMJ assembly, motor axons that invade nascent muscle fibers undergo presynaptic differentiation and accumulate synaptic vesicles containing acetylcholine and trophic factors. Concomitantly, the postsynaptic muscle domain shows increased expression of several proteins, including the nicotinic acetylcholine receptors, and the aggregation of these proteins—a hallmark of postsynaptic differentiation—in a restricted fraction of the muscle membrane to shape the nascent NMJ [1,2]. At the molecular level, NMJ assembly relies on trans-synaptic signaling triggered by secreted and extracellular matrix molecules from motor axons, skeletal muscle fibers, and tSCs [1,2]. For instance, the motor neuron-derived proteoglycan agrin plays major roles on the assembly and maintenance of postsynaptic acetylcholine receptors on the muscle membrane [3]. In this regard, cumulative evidence reveals that Wnt signaling pathways control crucial steps of NMJ synaptogenesis.

Wnt ligands (19 members in vertebrates) trigger different signalling pathways that play determinant roles on a wide range of biological scenarios lifelong [4]. In a simplified view, after Wnt binding to Frizzled (Fzd) receptors and the subsequent activation of the common intracellular effector Dishevelled (Dvl), three different pathways can be triggered. In the “canonical” Wnt pathway, the Wnt-dependent inhibition of the glycogen synthase kinase-3β (GSK3β) results in the intracellular accumulation and subsequent nuclear translocation of β-catenin that, along with TCF/Lef transcription factors, activates the expression of specific target genes [4]. Wnts also trigger two “non-canonical” pathways: the “planar cell polarity” pathway, which acts locally to modify the cytoskeleton through the small Rac and Rho GTPases, and a “Wnt calcium” pathway that regulates cell fate and cell movement by increasing intracellular Ca^2+^ levels [4]. Due to their high heterogeneity, the identification of specific Wnt responses depend on specific effectors for each cell type and biological context.

At the embryonic vertebrate NMJ, it has been shown that Wnt ligands secreted by different cellular sources, such as mesenchymal cells, muscle fibers, and motor axons, regulate different features of postsynaptic assembly [5,6,7,8,9,10,11]. In this regard, strong genetic evidence shows that the blockade of presynaptic (but not postsynaptic) secretion of Wnt ligands results in early NMJ defects, muscle weakness, and neurotransmission deficits [9]. According to this evidence, in situ hybridization studies show that the Wnt3 ligand is expressed by motor neurons at the time of NMJ formation [12]. In vitro, Wnt3 collaborates with agrin to induce AChRs clustering via a small GTPase-dependent, non-canonical Wnt signaling [7]. Although this evidence indicates that the motor axon terminal is a crucial source of Wnt ligands that help NMJ formation, such as Wnt3, two relevant issues remain unsolved: (i) is Wnt3 transported by motor axons and secreted at the NMJ, and (ii) does Wnt3 induce autocrine responses in motor neurons? Here, we have followed the transport, secretion, and function of recombinant Wnt3 overexpressed by transfected motor neuron-like NSC-34 cells and by developing chick motor neurons through in ovo electroporation. Functional experiments were performed in nerve-muscle co-cultures. Our findings led us to conclude that rather than affecting the morphological differentiation of motor neuron-like cells, neural-derived Wnt3 help postsynaptic differentiation at the vertebrate NMJs.

## 2. Materials and Methods

### 2.1. Chick Embryos

Fertilized chick eggs were incubated at 38 °C in a humidified incubator for specific time intervals. Embryos were staged according to Hamburger and Hamilton [13]. Experiments were conducted following the guidelines outlined in the Biosafety and Bioethics Manual of the National Commission of Scientific and Technological Research (CONICYT, Chilean Government) and the Ethics Committee of the University of Concepción.

### 2.2. Plasmids, Injection, and In Ovo Electroporation

We built the pSCP-nEGFP vector to allow the expression of two proteins using a single reading frame under the transcriptional control of the cytomegalovirus (CMV) promoter: the membrane-associated (via a myristoylation site) mCherry red fluorescent protein, which is separated of the coding sequence of a nuclear (via the nuclear localization sequence of the H2B histone) enhanced green fluorescent protein GFP (nEGFP) by a self-cleaving peptide (SCP) that allows the autocleavage of the two proteins at the amino acid position 18 of the SCP peptide between glycine 17 and proline 18, generating the two proteins with unaltered reading frames [14]. To build the pSCP-cWnt3HA plasmid, the coding sequence of chick Wnt3 (cWnt3) was amplified by RT-PCR from total RNA of HH27 chick embryos and cloned using NheI and HindIII restriction sites. Primer sequences were (5′ to 3′), for PCR1, cWNT3_Sall-FS: TATTAGTCGACATGGATTACCACCTGCTT, and cWnt3_link_HA_AS: CGGCACGTCGTACGGGTACTTGCAGGTA (expected product 1094 bp). For PCR2, cWNT3_Sall-F S and HA_XbaI-R AS: GCTCTAGACTACTACGCGTAGTCCGGC. The final expected product (1117 bp) was subcloned between the SalI and XbaI sites to replace the sequence of nEGFP in the pSCP-nEGFP plasmid. To add the HA epitope, a PCR reaction was performed using a primer that hybridizes to the 5′ region and add the coding nucleotides for the HA epitope. Rab11-mCherry was obtained from Addgene (https://www.addgene.org/55124/, 10 December 2021). BDNF-Venus was a generous gift from Dr. Orly Reiner’s Lab at the Department of Molecular Genetics of the Weizmann Institute of Science, Israel. The CD63-GFP plasmid was used and characterized as a marker of multi vesicular bodies, leading to the release of exosomes [15]. DNA injection and in ovo electroporation in the neural tube of chick embryos were performed as described [16] with some modifications. Briefly, the neural tube of HH15-17 embryos was injected with 1 mg/mL of plasmid DNAs coding for YFP, pSCP-cWnt3HA, or pSCP-nEGFP plus 0.1% Fast Green (Sigma) for visual monitoring of the injection. Electrodes were placed below the embryo (positive) and dorsal to the neural tube on the contralateral side (negative). Conditions used for electroporation were five square wave electrical pulses of 25 V, 50 ms pulse length, using the Ovodyne electroporator TSS20 (Intracel, Royston Herts, UK) and platinum electrodes. Following manipulation, the eggs were sealed with Parafilm (American National Can™, Greenwich, CT, USA) and returned to the incubator. After 48 h, embryos were fixed 12 h in 4% paraformaldehyde, and embedded in 30% sucrose. Samples were mounted in OCT (Sakura Finetek, Torrance, CA, USA) and cryosectioned at 30 μm.

### 2.3. Cell Culture

The neuroblastoma x spinal cord hybrid cell line NSC-34 [17] and the C2C12 cell lines were grown in DMEM (Invitrogen, Grand Island, NY, USA) supplemented with 10% fetal bovine serum (FBS) (HyClone, Logan, UT, USA) at 37 °C in 5% CO_2_. NSC-34 cells were induced to differentiate in Neurobasal medium (Invitrogen) without FBS for 24 h and 48 h. When indicated, conditioned media were prepared by transient transfection of NSC-34 expressing mouse Wnt3HA or control YFP and concentrated using methanol/chloroform. The level of secreted Wnt3HA was assessed by Western blots using anti-HA antibodies. For nerve-muscle co-cultures, C2C12 cells were seeded on glass coverslips coated with polyornithine/laminin, grown for 2 days, and differentiated for 5 days, as described [18]. Subsequently, undifferentiated NSC-34 cell previously transfected to express Wnt3HA or tdTomato were seeded on the myotube monolayer and subsequently differentiated for 24 h in Neurobasal medium. Co-cultured cells were treated with 200 pM neural agrin (R&D Systems) for 12–18 h at 37 °C and then stained with Alexa488-conjugated α-BTX (1 μg/mL) (Molecular Probes; 1:500) for 1 h at 37 °C, washed, and fixed in 4% paraformaldehyde for 20 min at 4 °C. Acetylcholine receptor aggregates were imaged through *z*-stacks series, which were collected using a Zeiss LSM700 confocal laser-scanning microscope (CMA Bio-Bio, Universidad de Concepcion). Morphologies were categorized into four different shapes, either as “clusters” [(i) microclusters (area < 5 μm^2^) or (ii) clusters (areas between 5 and 10 μm^2^)], or as “complex postsynaptic structures” [(iii) plaques (areas between 10 and 40 μm^2^) or (iv) pretzel-like (area > 40 μm^2^)]. Myotube surfaces were manually traced and their area was calculated using ImageJ. Data are expressed as the number of acetylcholine receptor aggregates per square micrometer of total myotube area and correspond to the average +/− SEM of 40–50 microscopic fields imaged using a 40× objective from three independent experiments performed in triplicate.

### 2.4. Transient Transfection

NSC-34 cells were incubated in OptiMEM medium (Invitrogen) and transfected using a Lipofectamine and Plus Reagent mix (Invitrogen), according to the indications of the manufacturer. The DNA/lipofectamine/PLUS ratio in the mixture was 0.5 μg/1.5 μL/1 μL with 0.4 μg plasmid DNA per well. NSC-34 cells were transfected with pCS2-Wnt3HA, pCS2-Fzd9HA, pCS2-mDvl1HA, and tdTomato. To study the subcellular distribution of Wnt3, NSC-34 cells were co-transfected in 1:3 ratio with the Wnt3HA plasmid along with CD63-GFP, BDNF-Venus, or Rab11-mCherry [15]. For luciferase reporter assays, the amounts of plasmid DNAs were TOPFlash 0.7 μg, pRL-SV40 70 ng.

### 2.5. Reverse Transcription-Polymerase Chain Reaction (RT-PCR)

Conventional RT-PCR to amplify endogenous Wnt3 was performed as described [19]. Control reactions were performed in the absence of reverse transcriptase to control for the presence of contaminant DNA. For amplification, the reaction mix was incubated 95 °C for 5 min, 95 °C for 30 s, 50 °C for 30 s, and 72 °C for 30 s for 35 cycles. Primers were Wnt3_S: TGACTCACATCATAAGGGGCCA, Wnt3_AS: CGGGCTAAGTACGCGCTCTTC (expected product 380 bp), mGAPDH_S: GGAGCCAAACGGGTCATCATCTC; GAPDH_AS: GAGGGGCCATCCACAGTCTTCT (expected product 233 bp). PCR products were separated and visualized after electrophoresis in 1.2% agarose gels.

### 2.6. Western Blot Analysis

Cells were lysed in PBS (cytosolic extract) or in Tris-HCl 50 mM, NaCl 100 mM, Triton X-100 0.5% *v*/*v* (total extract), pH 7.4, supplemented with a protease inhibitor cocktail (Sigma-Aldrich, St. Louis, MO, USA). For immunoblotting, 10 μg of total proteins or concentrated conditioned media were loaded per lane, fractionated by SDS-PAGE, transferred onto nitrocellulose membranes, and probed against rat anti HA (1:1000) (Roche Applied Science, Mannheim, Germany), mouse anti β-catenin (1:1000) (Santa Cruz Biotechnology, Santa Cruz, CA, USA), and goat anti β-actin (1:5000) (Santa Cruz Biotechnology, Santa Cruz, CA, USA) antibodies. Peroxidase-conjugated secondary antibodies (1:2000) (Jackson Immunoresearch, West Grove, PA, USA) were incubated for 2 h at room temperature. Reactions were developed with enhanced chemiluminescence according to the ECL Western blotting analysis system (Perkin Elmer, Waltham, MA, USA).

### 2.7. Immunohistochemistry, Immunofluorescence Microscopy, and Image Analyses

For immunohistochemistry, fixed chick embryo cryosections were washed with PBS containing 0.1% Tween-20, incubated with 0.15 mM glycine, pH 7.4, for 15 min at room temperature, and blocked with 1% BSA diluted in Tris phosphate buffer. NSC-34 cells grown on 12 mm glass coverslips were fixed in 4% paraformaldehyde for 30 min at 4 °C and permeabilized with 0.05% Triton X-100 in Tris-phosphate buffer. Primary antibodies were: rat anti HA (1:100; Roche Applied Science, Mannheim, Germany), mouse anti SV2 (1:200; form the Developmental Studies Hybridoma Bank, DSHB, Department of Biology, University of Iowa, IA, USA), and goat anti-MAP1B (1:150; Santa Cruz). Primary antibodies were incubated in 1% BSA diluted in Tris phosphate for 16 h at 4 °C. Corresponding Alexa488-, Alexa546 (Invitrogen)-, and Cy5 (Jackson Immunoresearch)-conjugated secondary immunoglobulins were incubated for 2 h at room temperature. Nuclei were counterstained with 30 μM DAPI (Molecular Probes, MA, USA) whereas microfilaments were labeled with Alexa546-phalloidin (1:1000, Life Technologies). Chick embryo NMJs were labeled with Alexa633-conjugated α-BTX (Molecular Probes; 1:500), along with the secondary antibodies. Samples were mounted in DAKO aqueous medium. Images were captured in a laser confocal LSM780 Zeiss microscope at the Center for Advanced Microscopy (CMA Bio-Bio) facility at Universidad de Concepción. In some experiments, mounted samples were visualized by Structural Illumination Microscopy (Zeiss ELYRA S1- SIM super-resolution laser microscope) with 63× (PlanApochromat 63×/1.4 Oil DIC M27) objective. The number of differentiated cells and the length of their neurites were determined in cells having at least one neurite with a minimum size equal to the cell soma diameter [19,20].

### 2.8. Statistical Analyses

Experiments with NSC-34 cells were performed at least three times by triplicate. In each condition, 10–15 microscopic fields per coverslip were quantified. Plots correspond to the mean ± SEM. One-way ANOVA was used for comparison among three or more groups followed by Bonferroni’s post hoc analysis for multiple comparisons between different groups. *p* < 0.05 was considered to indicate statistical significance.

## 3. Results

### 3.1. Wnt3 Is Expressed and Secreted by Differentiating Motor Neuron-like Cells

**Figure 1:**
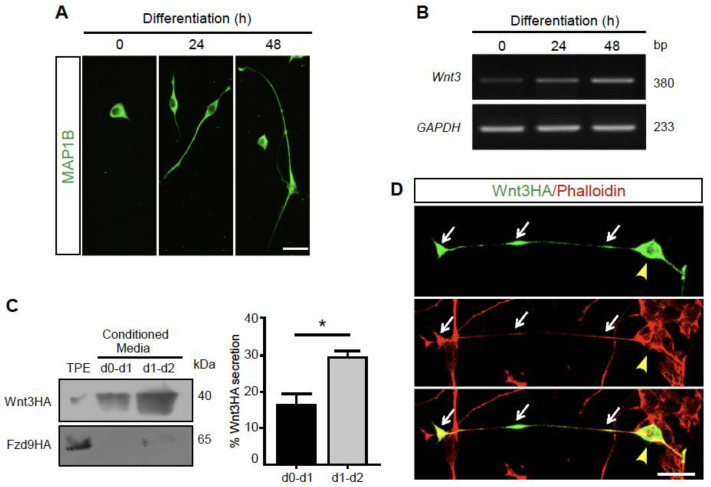
Wnt-3 is expressed and secreted by NSC-34 cells. (**A**) NSC-34 cells were cultured in growth medium and differentiated in Neurobasal medium for 24 or 48 h. Fixed cells were subjected to indirect immunofluorescence for MAP-1B. Bar, 30 µm. (**B**) Total RNA was obtained from NSC-34 cells at different times of differentiation and Wnt3 mRNA levels were analyzed by conventional RT-PCR. As loading control GAPDH was amplified. Negative control reactions were performed in the absence of reverse transcriptase (RT-). (**C**) NSC-34 cells were transfected with plasmids coding for Wnt3HA or Fzd9HA. Total protein extracts (TPE, obtained at 48 h) and concentrated conditioned media were subjected to Western blot using an anti HA antibody. A 40 kDa band was detected in TPE and conditioned media of NSC-34 cells expressing Wnt3Hal, whereas a 65 kDa was detected only in the TPE of cells expressing Fzd9HA. The panels on the left show representative band intensity. The plot on the right shows the average ± SEM of the band quantification from three independent experiments (* *p* < 0.05; *t*-test) (**D**) NSC-34 cells were transfected with Wnt3HA and stained with an anti HA antibody plus the microfilament marker phalloidin. Arrowheads and arrows indicate Wnt3HA distribution in the cell soma, as well as in neurites and growth cone tips, respectively. Bar, 30 µm.

In our in vitro studies, we used the NSC-34 cell line, a well-defined and reproducible in vitro model of vertebrate motor neurons, as they are cholinergic, generate action potentials, and, when co-cultured with differentiated myotubes, form neuromuscular contacts [17]. When transferred to neurobasal medium, NSC-34 cells undergo a time-dependent differentiation process towards a neuronal phenotype characterized by neurite extension [19,20] (Figure 1A). We first analyzed the expression of endogenous Wnt3 in NSC-34 cells by conventional RT-PCR (Figure 1B). We found that differentiating NSC-34 cells express the Wnt3 transcript. Compared with the band intensity of the housekeeping gene GAPDH, Wnt3 expression is upregulated as neuronal differentiation proceeds (Figure 1B).

As a first hint to follow Wnt3 secretion, undifferentiated NSC-34 cells were transfected with a plasmid coding for mouse Wnt3 fused to the HA tag (Wnt3HA) and differentiated for 48 h. Conditioned media were collected from the onset of differentiation after 24 h (d0-d1) or 48 h (d1-d2), concentrated, and subjected to Western blot analyses using an anti HA antibody. Results in Figure 1C (left panel) show that Wnt3HA is accumulated in the conditioned media of differentiating NSC-34 cells. Quantification using the band intensity of a 1/50 fraction of a total protein extract (TPE) as a standard showed that 16.3 and 28.9% of overexpressed Wnt3HA reach the conditioned media after 24 and 48 h, respectively (Figure 1C, right panel). To confirm that the presence of Wnt3HA in conditioned media is due to secretion and not to cell death or any other unspecific effect, NSC-34 cells were transfected with a plasmid coding for the Wnt receptor Frizzled-9 fused to HA (Fzd9HA; [21]). Western blot analyses show almost undetectable levels of Fzd9 in conditioned media at 24 and 48 h (Figure 1C, left panel). Protein extracts from control NSC-34 cells transfected with an empty pBlueScript plasmid showed no HA signal (data not shown).

To begin to analyze the subcellular distribution of Wnt3, we performed immunocytochemical detection in transfected NSC-34 cells using the anti HA antibody (Figure 1D). We found that Wnt3HA distributes in cell bodies and neurites of differentiated NSC-34 cells (Figure 1D). The increased expression of Wnt3 upon differentiation as well as the secretion and distribution of Wnt3HA in the cell soma and neurites support previous findings, suggesting that Wnt3 is transported and secreted by motor neurons [7].

### 3.2. Overexpressed Wnt3 Is Transported by Motor Neurons in a Vesicular-like Pattern

**Figure 2:**
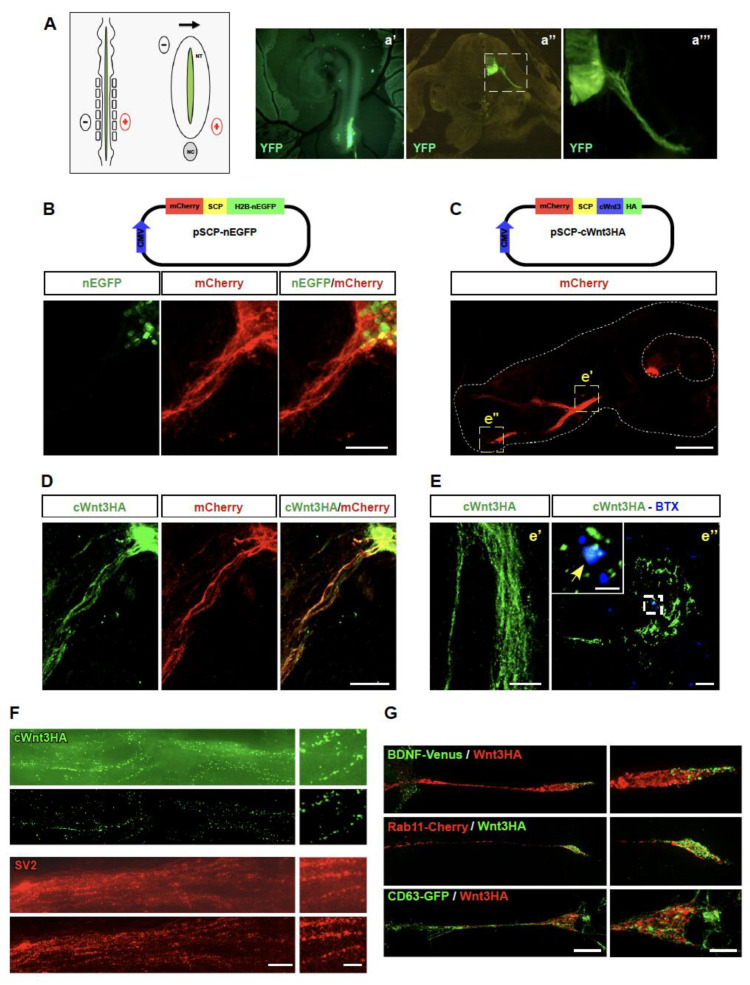
Overexpressed Wnt3 distributes in a vesicular-like pattern along motor axons and reaches the neuromuscular junction area in the limb region of developing chick embryos. (**A**) Schematic diagrams showing the in ovo electroporation approach. The DNA is microinjected into the central canal of the neural tube and electrodes are positioned to target motor neuron somas in the ventral spinal cord. The arrow indicates DNA migration. NT: neural tube; NC: notochord). (**a′**–**a‴**) Panels show a HH16 chick embryo electroporated to express YFP. Positive YFP signal (green) distributes in somas and axons at the right ventral neural tube, as visualized in (**a′**) the whole embryo, as well as in transversal sections at (**a″**) low (Bar, 100 µm) and (**a‴**) high magnification (Bar, 50 µm). (**B**) The ventral neural tube of HH16 chick embryos was electroporated with the pSCP-nEGFP plasmid. After 24 h, transversal sections show nuclear EGFP and membrane-anchored mCherry expression in ventral motor neurons (Bar, 30 µm). (**C**) HH16 chick embryos were electroporated with the pSCP-cWnt3HA plasmid. A low magnification image shows mCherry distribution in motor neurons (Bar, 500 µm). (**D**,**E**) Immunohistochemical detection reveals that cWnt3HA and mCherry are co-expressed in cell bodies and along motor axons. (**E**) Higher magnification images show that cWnt3HA distributes in a punctate pattern along motor axons (**e′**) as well as in their distal hind limb portion (**e″**), where it co-localizes with muscle postsynaptic acetylcholine receptor aggregates (blue). The images are representative of three independent experiments (Bars, 30 µm in e′, 10 µm in (**e″**), 2.5 µm in the inset of (**e″**)). (**F**) Transversal cryosections of electroporated chick embryos were analyzed with a Zeiss Elyra S1 microscope, equipped for SIM super-resolution (Bar, 10 µm). For each marker, the upper panel displays brightfield plus SIM, whereas the lower panel is the SIM image alone. Panels on the right are magnified images of the left panel images (Bar, 10 µm). (**G**) NSC-34 cells were co-transfected with a plasmid coding for Wnt3HA along with either BDNF-Venus, Rab11-mCherry, or CD63-GFP. Cells were differentiated for 24 h and subjected to indirect immunofluorescence with antibodies against HA. Images were obtained by SIM super-resolution (Bar, 5 µm). Panels on the right are magnified images of the neurite tips in the left panel images (Bar, 2.5 µm). The images are representative of three independent experiments.

We next sough to analyze the transport and secretion of Wnt3 in vivo. With this aim, we setup in ovo electroporation experiments in the neural tube of developing chick embryos [16,22]. Control experiments in HH21 chick embryos electroporated to overexpress the yellow fluorescent protein (YFP) show that our procedure specifically targets motor neurons, as revealed by their big soma size and because their axons leave the ventral spinal cord (Figure 2A). As a tool to analyze the behavior of Wnt3 in vivo, the coding sequence of chick Wnt3 (cWnt3) was cloned into the pSCP-nEGFP plasmid (Figure 2B). This unique reading frame was designed to allow the simultaneous translation of membrane-targeted mCherry separated by a “self-cleaving peptide” of a nuclear-targeted EGFP (nEGFP), in the same cells [14]. Control experiments showed that both fluorescent proteins are expressed separately after in ovo electroporation of spinal cord motor neurons (Figure 2B). Next, the coding sequence of cWnt3 expressing a C-terminus HA tag was subcloned into the pSCP-nEGFP by replacing the nEGFP sequence. The resulting pSCP-cWnt3HA plasmid was electroporated in the neural tube of developing chick embryos (Figure 2C). A low magnification image shows that mCherry is expressed in the cell soma and motor axons towards developing hindlimbs (Figure 2C). Anti HA immunocytochemical detection confirms that cWnt3HA and mCherry are detected in the cell bodies and the proximal axonal regions of developing chick motor neurons (Figure 2D). Interestingly, higher magnification images show that cWnt3HA distributes in a punctate pattern along the motor axons (Figure 2E, e′) and reaches the most distal hind limb region, where the anti HA signal co-localizes with muscle postsynaptic domains, as revealed by staining of acetylcholine receptor aggregates with alpha-bungarotoxin (Figure 2E, e″).

As a first approach to discriminate if Wnt3 distribution corresponds to a vesicular pattern, cryosections of chick embryos electroporated to express cWnt3HA were analyzed through structured light super-resolution (SIM) microscopy (Figure 2F). SIM images reveal that cWnt3HA distributes in discrete puncta all along motor axons (Figure 2F), a feature that can be further improved by avoiding the brightfield layer (lower images in Figure 2F). Even though double staining was not possible due to technical limitations, in adjacent chick embryo sections we found that the endogenous synaptic vesicle 2 protein (SV2) follows a comparable distribution and puncta size to cWnt3HA staining (Figure 2F). To further study the vesicular-like distribution of Wnt3, NSC-34 cells were transfected to co-express Wnt3HA along with BDNF-Venus to identify secretory and dense core vesicles [23,24] or with Rab11-mCherry to reveal recycling endosomes and axonal transcytotic vesicles [25,26]. SIM microscopy revealed a poor co-distribution of Wnt3HA with both vesicular markers (Figure 2G). Considering that the *Drosophila* Wnt homologue Wingless (Wg) is secreted by presynaptic motor axons in exosomes at the fly NMJ [27,28], we co-transfected NSC-34 cells to co-express Wnt3HA along with the late endosome and multivesicular bodies marker CD63-GFP, a protein that is also abundant in exosomes [15]. Our results show that Wnt3HA does not co-localize with multi vesicular bodies in differentiated NSC-34 cells (Figure 2G). Our findings reveal that Wnt3 is transported towards the neuromuscular synapse in a vesicular-like pattern by still unidentified secretory vesicles.

### 3.3. Motor Neuron-Secreted Wnt3 Induces Postsynaptic Assembly

**Figure 3:**
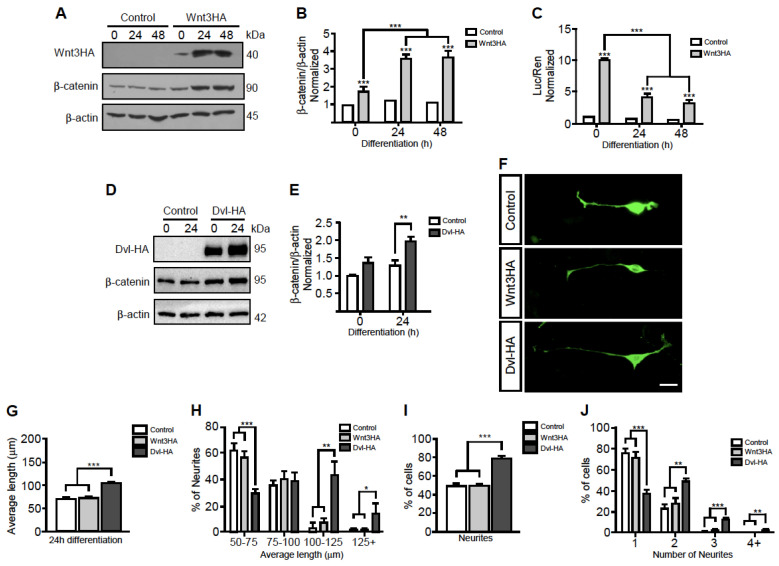
NSC-34 cells overexpressing Wnt3HA do not modify their morphological differentiation but induce acetylcholine receptor aggregation on co-cultured myotubes. (**A**) Total protein extracts were obtained from NSC-34 cells transfected to overexpress Wnt3HA at 0, 24, and 48 h of differentiation. Control cells were transfected with empty pBluescript plasmid. Protein extracts were subjected to Western blot analyses using anti HA, anti β-catenin, and anti β-actin antibodies, the latter used as loading control. (**B**) The plot represents the quantification of the band intensity ratio between β-catenin and β-actin expressed as the average ± SEM of three independent experiments. (**C**) NSC-34 cells were co-transfected with Wnt3HA and the Wnt reporter Topflash plus the pRLSV40 Renilla control plasmids and subsequently differentiated for the indicated times. The graph shows the normalized reporter gene activity (Luciferase Topflash/Renilla; Luc/Ren). Data are expressed as the average ± SEM of three independent experiments performed by quadruplicate. (**D**) Total protein extracts from NSC-34 cells overexpressing Dvl-HA at 0 and 24 h of differentiation were subjected to Western blot analyses using anti HA, anti β-catenin, and anti β-actin antibodies. Control cells were transfected with a plasmid coding for yellow fluorescent protein (YFP). (**E**) The plot represents the quantification of the band intensity ratio between β-catenin and β-actin expressed as the average ± SEM of three independent experiments. (**F**) NSC-34 cells transfected to express tdTomato, Wnt3HA, or Dvl-HA were differentiated for 24 h, fixed, and stained with an anti MAP1B antibody (Bar, 20 µm). Morphological parameters were measured using ImageJ. These include (**G**) the average length of neurites, (**H**) the distribution of neurite length, (**I**) the proportion of cells bearing neurites, and (**J**) the percentage of cells having different numbers of neurites. Plots are the average ± SEM of three independent experiments (* *p* < 0.05; ** *p* < 0.01; *** *p* < 0.001; ANOVA).

We finally aimed to study the function of Wnt3 secreted by motor neurons. As signaling pathways triggered by Wnt ligands affect diverse parameters of neuronal differentiation [29,30], we next analyzed if Wnt3 overexpression could: (i) activate Wnt pathways in NSC-34 cells, and/or (ii) affect their morphological differentiation. To analyze the canonical Wnt pathway, protein extracts from differentiating NSC-34 cells expressing Wnt3HA were subjected to Western blot using an anti β-catenin antibody, whereas an anti β-actin antibody was used as loading control (Figure 3A). Band intensity quantification shows that Wnt3 overexpression induces β-catenin accumulation during the differentiation of NSC-34 cells (Figure 3B). To correlate these results with Wnt-dependent transcription, we also expressed the Wnt reporter Topflash plasmid in NSC-34 cells (Figure 3C). We found that Wnt3 overexpression strongly induced the Wnt reporter gene at all differentiation stages of NSC-34 cells, being statistically higher in undifferentiated cells (Figure 3C). Together, these findings show that Wnt3 activates canonical Wnt signaling in motor neuron-like cells.

To analyze the possibility that Wnt3 could exert autocrine effects on motor neurons, we analyzed morphological parameters of neuronal differentiation in NSC-34 cells expressing Wnt3HA. Negative control cells were transfected with the YFP plasmid. As a positive control, we transfected NSC-34 cells with a plasmid coding for Dishevelled-1 (Dvl) fused to the HA tag, as Dvl acts as a general activator of Wnt responses [31]. Indeed, control Western blot experiments (Figure 3D) showed that Dvl overexpression induces β-catenin accumulation in differentiated NSC-34 cells, as Wnt3 does (Figure 3E). Microscopic analyses showed that whereas cells in all conditions extend neurites, no discernible or significant effects were observed by Wnt3 overexpression; in turn, Dvl-1 overexpression resulted in cells bearing longer neurites (Figure 3F). Quantification shows that the average length and the proportion of cells bearing longer neurites were significantly increased by DvlHA, but not by Wnt3HA overexpression (Figure 3G,H). A similar response was observed regarding the proportion of cells projecting neurites and when the proportion of cells extending two or more neurites was quantified (Figure 3I,J). Therefore, despite the fact that motor neuron-like NSC-34 cells modulate their morphological differentiation upon Wnt activation, the overexpression of the Wnt3 ligand and the subsequent activation of canonical Wnt signaling does not significantly affect their neuronal differentiation.

**Figure 4:**
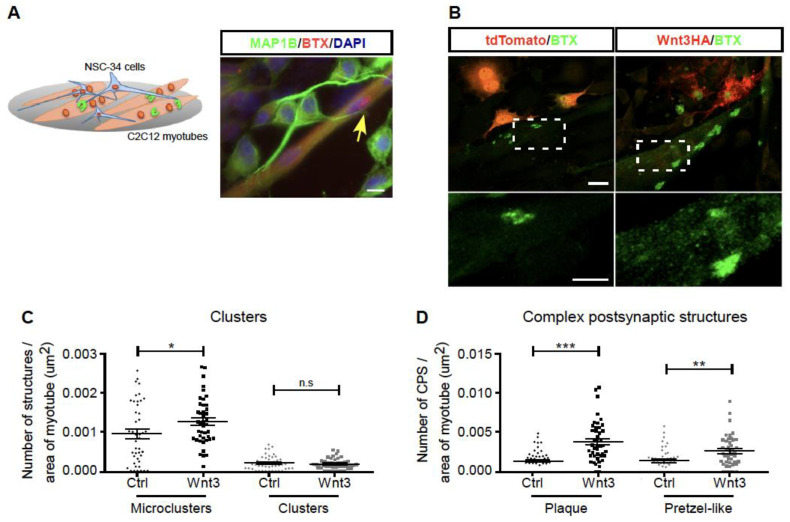
NSC-34 cells overexpressing Wnt3 enhance acetylcholine receptor clustering on co-cultured myotubes. (**A**) Scheme of the nerve-muscle co-cultures using C2C12 myotubes seeded onto polyornithine/laminin-coated dishes and differentiated NSC-34 cells seeded on top. The panel shows a control co-culture stained with an anti MAP1B antibody, along with Alexa594-α-bungarotoxin (BTX). Nuclei were counterstained with DAPI. (**B**) NSC-34 cells expressing tdTomato (control) or Wnt3HA were seeded on top of C2C12 myotubes and differentiated for 24 h. Acetylcholine receptor aggregates were labeled with Alexa488-α-bungarotoxin (BTX) (Bar, 20 µm). Lower panel images are high magnifications of the dotted line squares (Bar, 10 µm). Acetylcholine receptor aggregates were quantified and categorized into four different shapes, according to their area, as follows: (**C**) “clusters” which were separated into microclusters or clusters, or (**D**) “complex postsynaptic structures”, which were subdivided into plaques or pretzel-like structures. Myotube surfaces were manually traced and their area was calculated using ImageJ. Data are expressed as the number of acetylcholine receptor aggregates per myotube area. Plots are the average ± SEM of 40–50 microscopic fields imaged at a 400× magnification from three independent experiments performed in triplicate (* *p* < 0.05; ** *p* < 0.01; *** *p* < 0.001; *t*-test).

It has been previously shown that the exposure of myotubes of the C2C12 cell line to Wnt3 induces the clustering of acetylcholine receptors, a main feature of postsynaptic differentiation at the NMJ [7]. As an alternative strategy to approach this idea in culture conditions closer to a physiological situation, we setup nerve-muscle co-cultures. With this aim, C2C12 myoblasts were seeded onto polyornithine/laminin-coated dishes to induce the formation of complex postsynaptic structures, similar to those observed in vivo [18,32]. Once differentiated into myotubes, NSC-34 cells were seeded on top of differentiated myotubes for 24 h and subsequently treated with neural agrin (Figure 4A). Control experiments show that although neurites of differentiated NSC-34 cells contact myotubes, the formation of acetylcholine receptor aggregates is not locally stimulated in our culture conditions, and complex postsynaptic structures are likely induced only by the laminin coating (Figure 4A). A similar result was observed when differentiated myotubes were co-cultured with NSC-34 cells expressing the tdTomato fluorescent protein (Figure 4B). Remarkably, when co-cultured with Wnt3-expressing NSC-34 cells, myotubes assemble acetylcholine receptor microclusters on their surface, a feature that was particularly evident in myotubes allocated in the vicinity of neurites, which display a punctate distribution of Wnt3 (Figure 4B, lower panel). In addition, bigger and brighter complex postsynaptic structures were visible in cultures containing Wnt-expressing NSC-34 cells, as compared with controls (Figure 4B). Quantification shows that NSC-34 cells overexpressing Wnt3 stimulate the number of acetylcholine receptor microclusters (area < 5 μm^2^) (Figure 4C), as well as the abundance of complex postsynaptic structures, either plaques (areas between 10 and 40 μm^2^) or pretzel-like (area > 40 μm^2^) (Figure 4D). Altogether, our findings support the notion that presynaptic Wnt3 is transported and secreted by motor neurons to help postsynaptic differentiation in nascent NMJs.

## 4. Discussion

At the vertebrate NMJ, experimental strategies aimed to cause a general inhibition of all Wnt cascades have shown that they regulate crucial steps of NMJ development. For instance, embryonic NMJs of mice null for the general Wnt effector Dvl display abnormal distribution [7]. Consistently, exposure of chick embryo forelimbs to the broad Wnt antagonist Secreted Fzd-related protein-1 (Sfrp1) decreases early AChR clustering in nascent muscle fibers [7] and similar results were obtained after Sfrp4 injection in developing mice [8].

A crucial feature in studies involving the effect of Wnt ligands on NMJ synaptogenesis is the identification of the cellular source(s) of Wnt secretion. In this regard, it has been shown that Wnt ligands secreted by mesenchymal cells regulate the early aneural clustering of AChRs on nascent muscle fibers in zebrafish [5,6], and a similar mechanism likely occurs in mice [8,10]. In turn, developing skeletal muscle fibers express Wnt3a, a ligand that inhibits agrin-dependent AChR clustering in vitro and disperses AChRs in vivo through canonical pathway activation [11]. Interestingly, genetic loss-of-function of the Wnt ligand secretion mediator Wls specifically in motor neurons (but not in muscles or Schwann cells) resulted in severe defects on NMJ organization, with consequent muscle weakness and neurotransmission deficits [9]. According to the key role of presynaptic Wnt secretion for NMJ organization, in situ hybridization studies show that the Wnt3 ligand is expressed by motor neurons at the time of NMJ formation [12]. Functionally, in vitro experiments reveal that Wnt3 collaborates with agrin to induce AChRs clustering via a small GTPase-dependent non-canonical Wnt signaling [7].

Based on this evidence, we aimed to experimentally determine the cellular behavior of overexpressed Wnt3 in cultured motor neuron-like cells and in vivo motor neurons following a similar experimental strategy of studies following the traffic and secretion of neural agrin, a major presynaptic-derived NMJ organizer [33]. On the one hand, we considered that the *Drosophila* Wnt orthologue Wingless (Wg) is secreted in exosomes associated with the Wnt-binding protein Evenness Interrupted (Evi) at the fly NMJ, via a mechanism dependent on Rab11 and Syntaxin1A (Syx1A) [27,28]. On the other hand, it has been shown that the Wnt5a ligand is distributed in a vesicular pattern in axons of hippocampal neurons in culture and accumulates in presynaptic terminals [34]. Our results show that Wnt3 is also distributed in a vesicular-like pattern in NSC-34 cells that resembles the distribution of the SV2 marker, a membrane glycoprotein localized in neuronal secretory vesicles [35]. Our findings show that the vesicular-like distribution of Wnt3HA does not colocalize with the exosome marker, nor with BDNF dense core vesicles (DCV) or with Rab11, which opens the possibility that the transport of Wnt3 secretory vesicles along motor axons could be driven by other monomeric GTPases that are known to mediate the axonal transport of different vesicles, such as Rab2, Rab27a, or Rab3 [36,37,38]. In addition, future studies should consider other general markers of secretory vesicles, including SNARE proteins such as synaptobrevin 2 and synaptotagmins, as these proteins likely control the secretion of different subsets of secretory vesicles in axons [24,39,40]. Interestingly, a recent report combines elegant transmission electron microscopy and Western blots experiments in isolated vesicles to show that Wnt-3a, -5a, and -7a are secreted in CD63-positive small extracellular vesicles from hippocampal HT-22 neurons [41]. In spite of the still missing identification of its precise secretory pathway, our SIM microscopy results combined with our secretion studies in transfected motor neuron-like cells support the idea that Wnt3 is transported by vesicles and secreted by motor neurons at the developing NMJ.

Based on the abundant evidence showing that Wnt ligands induce neuronal differentiation ([29,30], and the references therein), we analyzed a potential autocrine effect of Wnt3 expression and secretion on the morphological differentiation of motor neuron-like NSC-34 cells. We found that although Wnt3 activated β-catenin-dependent canonical Wnt signaling, this ligand does not affect the morphological differentiation of NSC-34 cells. In cultures of developing spinal cord neuronal precursors, both Wnt3 and Wnt-3a induce the expression of neuronal markers and neurite outgrowth through β-catenin-dependent transcription [42]. One main difference of these studies with our current results could be that spinal cord precursor cultures are enriched in interneurons rather than in motor neurons [43]. Regarding motor neuron-like cells, we have previously studied Wnt signaling in NSC-34 cells stably expressing a G93A-mutated form of human superoxide dismutase 1 (hSOD1) as a model of amyotrophic lateral sclerosis motor neurons [20,44]. In control NSC-34 cells expressing wild-type hSOD1, canonical Wnt pathway activation through GSK3β inhibition using lithium chloride slightly decreases the proportion of cells bearing neurites, whereas the more specific GSK3β inhibitor Andrographolide did not have a significant effect on neuronal differentiation [44]. In addition, none of the GSK3β inhibitors affect neurite length [44]. Interestingly, our findings show that both Wnt3 and Dvl induce β-catenin accumulation in NSC-34 cells, but only Dvl overexpresssion induced their morphological differentiation, suggesting that Wnt pathways other than canonical Wnt signaling likely command neurite extension in NSC-34 cells. It is worth asking whether canonical Wnt signaling affects motor neuron differentiation? It is relevant to mention that NSC-34 cells expressing mutant hSOD1 display decreased Wnt-dependent transcription, which correlated with reduced expression of the motor neuron transcription factor HB9 [20]. From these findings, we speculate that Wnt3 activation of canonical Wnt signaling might be related to the transcription of specific motor neuron genes rather than with morphological parameters. Additionally, we conclude that the increased morphological differentiation of NSC-34 cells overexpressing Dvl could be related to the ability of Dvl to trigger local non-canonical pathways that control cytoskeleton organization to induce the extension of neuronal projections [45,46,47]. Future comparative studies will help to elucidate the specific contribution of different Wnt pathways to the presynaptic differentiation of motor neurons at developing NMJs.

Our previous findings showed that Wnt3 obtained from conditioned media of transfected heterologous cells has the ability to collaborate with agrin to induce AChRs clustering in C2C12 myotubes [7]. In our current studies, we conducted experiments to better mimic a physiological condition by: (i) using cultures of C2C12 myotubes on laminin-coated dishes that resemble the NMJ extracellular environment and induce the formation of complex postsynaptic structures [18,32], and (ii) establishing co-cultures of these myotubes with NSC-34 cells overexpressing Wnt3. We found that when co-cultured with Wnt3-expressing NSC-34 cells in the presence of agrin, C2C12 myotubes exhibited not only an evident increase in acetylcholine receptors microclusters but also formed bigger complex postsynaptic structures. Indeed, NSC-34 cells overexpressing Wnt3 stimulate the number of acetylcholine receptor microclusters, as we previously showed on C2C12 myotube cultures treated with Wnt3-enriched conditioned media [7]. Remarkably, Wnt3-expressing NSC-34 cells also increased the abundance of complex postsynaptic structures assembled onto laminin-coated surfaces, either plaques or pretzel-like aggregates. These results show that, along with enhancing the acetylcholine receptor clustering ability of the neural-derived protein agrin [7], Wnt3 could also potentiate the aggregating effect of laminin, a key extracellular matrix regulator of the formation of mature-like postsynaptic apparatuses [18,32]. These results further highlight the potential physiological role of motor neuron-derived Wnt3 on postsynaptic assembly and maintenance at the vertebrate NMJ.

Identifying the specific contribution of Wnt3 alone or in combination with agrin and/or laminin to the assembly of complex postsynaptic structures will provide valuable information regarding the potential role of presynaptic Wnt secretion on the maintenance of mature NMJs. This issue is particularly relevant considering the cumulative evidence showing that the expression of Wnt proteins is markedly altered in conditions affecting the NMJ integrity, such as amyotrophic lateral sclerosis or other motor diseases [48,49]. In this regard, in cultured C2C12 myotubes, Wnt3 positively regulates the expression of Lrp4 [50], an agrin co-receptor that plays crucial roles on the embryonic formation and maintenance of mature NMJs [51]. Remarkably, functional alterations of Lrp4 using blocking antibodies or specific mutations, both observed in human patients, induce Myasthenia gravis and a form of Congenital myasthenic syndrome, respectively [52,53]. Therefore, the dissection of the mechanisms and cell sources of Wnt ligands expression and secretion could provide powerful targets to be manipulated in future therapeutical strategies to maintain synaptic structure and function in conditions leading to NMJ decline.

## 5. Conclusions

We conclude that Wnt3 is transported along motor axons in a vesicular-like pattern and reaches their most distal muscle portion, where it is likely secreted. Even though Wnt pathways are able to modify presynaptic differentiation, our results also reveal that motor neuron-secreted Wnt3 performs specific muscle postsynaptic effects at the NMJ, whereas Wnt3 secretion did not modify the morphological differentiation of motor neuron-like cells. Our findings in nerve-muscle co-cultures lead us to speculate that, once secreted, Wnt3 collaborates with other nerve- and muscle-secreted signals to help postsynaptic differentiation on the muscle surface.

## Data Availability

Requests for plasmids and detailed methods used in the present study should be directed to the corresponding author.
